# Nonsense variant of *NR0B1* causes hormone disorders associated with congenital adrenal hyperplasia

**DOI:** 10.1038/s41598-021-95642-y

**Published:** 2021-08-09

**Authors:** Da-Bei Fan, Li Li, Hao-Hao Zhang

**Affiliations:** 1 Endocrine Department, The First Affiliated Hospital of Zhengzhou University, Zhengzhou, 450052 China; 2 Ophthalmologic Center, The First Affiliated Hospital of Zhengzhou University, Zhengzhou, 450052 China

**Keywords:** Endocrine system and metabolic diseases, Cell biology, Molecular biology

## Abstract

Congenital adrenal hyperplasia (CAH) is a rare X-linked recessive inherited disease that is considered a major cause of steroidogenesis disorder and is associated with variants or complete deletion of the *NR0B1* gene. The DAX-1 protein (encoded by *NR0B1*) is a vertebrate-specific orphan nuclear receptor and is also a transcriptional factor for adrenal and reproductive development. CAH usually causes adrenal insufficiency in infancy and early childhood, leading to hypogonadotropic hypogonadism in adulthood; however, few adult cases have been reported to date. In this study, we examined a Chinese family with one adult patient with CAH, and identified a putative variant of *NR0B1* gene via next-generation sequencing (NGS), which was confirmed with Sanger sequencing. A novel nonsense variant (c.265C>T) was identified in the *NR0B1* gene, which caused the premature termination of DAX-1 at residue 89 (p.G89*). Furthermore, mutant *NR0B1* gene displayed a partial DAX-1 function, which may explain the late pathogenesis in our case. Additionally, qPCR revealed the abnormal expression of four important genes identified from ChIP-seq, which were associated with energy homeostasis and steroidogenesis, and were influenced by the DAX-1 mutant. In addition, hormone disorders can be caused by DAX-1 mutant and partially recovered by siRNA of *PPARGC1A*. Herein, we identified a novel nonsense variant (c.265C>T) of *NR0B1* in a 24-year-old Chinese male who was suffering from CAH. This mutant DAX-1 protein was found to have disordered energy homeostasis and steroidogenesis based on in vitro studies, which was clinically consistent with the patient’s phenotypic features.

## Introduction

There is growing evidence that congenital adrenal hypoplasia (CAH) is primarily caused by adrenal insufficiency and poses a serious threat to males^1^. CAH is a rare monogenic disorder that affects approximately 1/15,000 of live births. Recent advancements in sequencing techniques and bioinformatic tools have expanded our understanding of the molecular genetic basis and the variant spectrum of CAH. Various novel variants associated with CAH have been reported to date, suggesting that the identification of gene variants in CAH could be a promising approach to diagnose CAH^2–4^. Patients with this disorder usually show signs and symptoms of adrenal insufficiency in early infancy or throughout childhood. In contrast, recent studies have reported that patients with X-linked CAH show symptoms during late adolescence or even in adulthood^5–7^. Normally, the treatment for CAH includes lifelong glucocorticoid and mineralocorticoid replacement therapy^8,9^.

Most CAH cases follow an autosomal recessive or X-linked traits of inheritance^1,5^. Specific variants of the *NR0B1* gene (the coding gene for the DAX-1 [dosage sensitive sex-reversal, adrenal hypoplasia locus on the X-chromosome, gene 1] protein), including point variants and delete variants, have been shown to cause X-linked CAH^10,11^. DAX-1 is an orphan nuclear hormone receptor that regulates adrenal development and functions as a dominant negative regulator of other nuclear receptors^11^. Moreover, DAX-1 encodes a protein of 470 amino acids, which is a member of the orphan nuclear hormone receptor superfamily and acts as a transcriptional repressor of genes involved in the steroidogenesis pathway^12,13^. The duplication of *NR0B1* can cause a male-to-female sex reversal phenotype, while variants in *NR0B1* lead to CAH associated with hypogonadotropic hypogonadism; this highlights the crucial role of DAX-1 in sex determination and gonadal development^14,15^. However, the biological role of DAX-1 mutant in patients with CAH remains unclear despite these aforementioned studies. A missense variant of DAX-1 (p.V385L) has been reported to be associated with nonobstructive azoospermia, indicating the important role of DAX-1 in spermatogenesis^16^. Numerous variants associated with CAH have been identified in the human *NR0B1*; few in-depth studies have uncovered the intricate molecular mechanism of the role of specific *NR0B1* variants in CAH development.

In the present study, we clinically examined a 24-year-old male affected with CAH from a three-generation family and identified a novel nonsense variant of the *NR0B1* gene via next-generation sequencing (NGS). Furthermore, ChIP-seq analysis was performed to dissect several target genes such as *CARTPT*, *PPARGC1A*, *PDE4B*, and *PDE4D*, which led energy homeostasis and steroidogenesis to be associated with CAH for the first time. In addition, secreted hormones and related gene expression changes influenced by the DAX-1 mutant, which were partially recovered by silencing of PPARGC1A, implied that this *NR0B1* variant caused CAH, providing a preliminary understanding of the potential molecular mechanism driving CAH. Taken together, these findings provide a preliminary understanding of the potential molecular mechanism driving CAH and significantly advance our molecular understanding of the varietal spectrum of *NR0B1* in the context of CAH.

## Materials and methods

### Clinical examination

**Figure 1:**
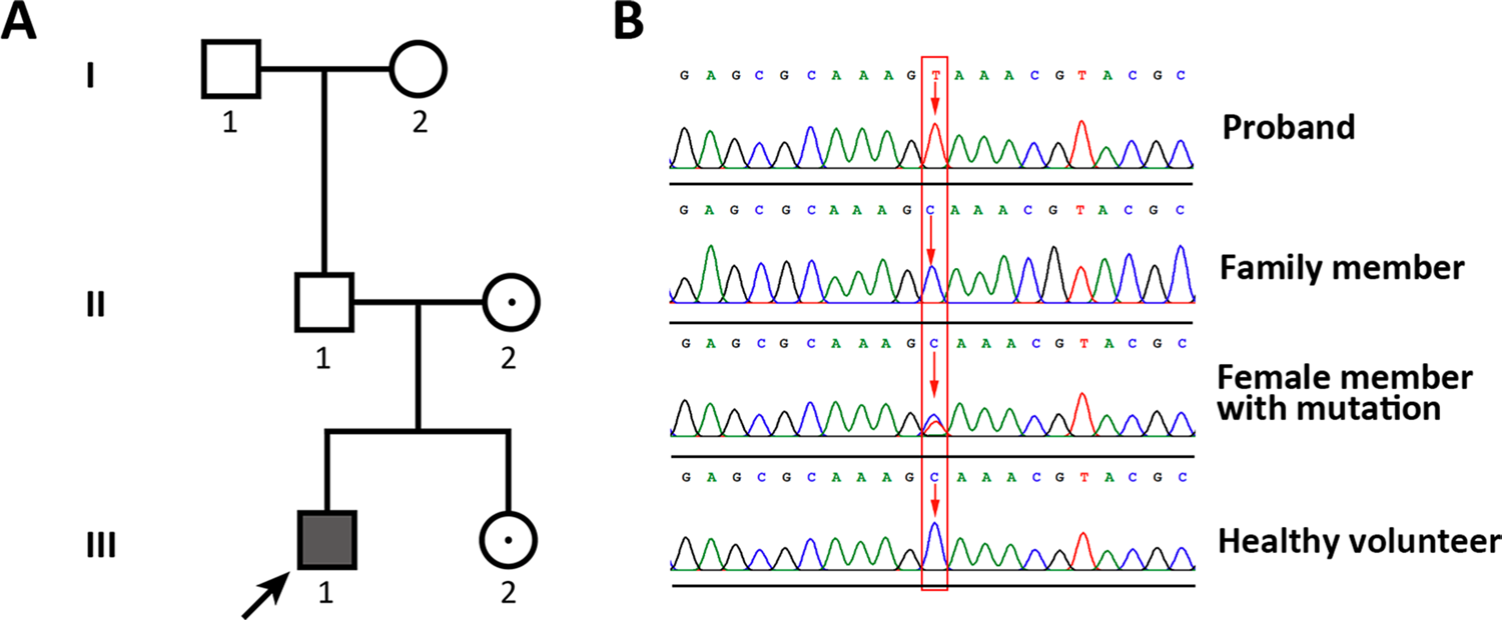
Family pedigree and variant screening analysis. (**A**) Family history of the three-generation family. Dark symbols represent the affected members, and clear symbols represent the unaffected members, among them, a dot in a clear symbol represents an unaffected member with a heterozygous gene variant. A square indicates a male and a circle indicates a female. (**B**) Screening of the variant site in the family and healthy volunteers is shown in (**A**) by sequencing analysis. The red frame indicates the variant site.

A Chinese family of Han ethnicity from Henan Province was identified and the family members were recruited for this study from the First Affiliated Hospital of Zhengzhou University (Fig. 1A). One 24-year-old male (46,XY karyotype) with CAH was physically and clinically examined to determine physical and clinical symptoms. His height and weight were 172.7 cm and 41.2 kg, respectively, and he appeared slightly emaciated. In addition, his blood pressure was 114/68 mmHg without medication. The medical history of the affected family members was also recorded. At least 100 unrelated healthy participants without endocrine diseases or other clinical diseases from Henan Province were chosen as healthy controls. Venous blood samples were collected from the proband and his family members and placed in tubes containing ethylenediaminetetraacetic acid (EDTA) for DNA isolation and NGS sequencing.

### NGS and Sanger sequencing

Blood DNA was isolated from venous blood samples collected from patients using the QIAamp DNA Blood Mini Kit (Qiagen, USA) following standard protocols and then sent to BGI (Shenzhen, China) for exonic capture and panel re-sequencing. Library preparation was performed following standard protocols as described previously^17^. NimbleGen custom arrays, which enriched the exonic sequences of endocrine-related genes and other genes that have been shown to cause related steroidogenesis disorder, were used for the capture procedure. Furthermore, paired-end sequencing for 100-bp reads was performed on an Illumina HiSeq2000 platform (Illumina, USA).

Raw sequence reads were first filtered using Trim-Galore to export clean data, which was subsequently aligned to the human genome using the BWA program^17^. The quality of these clean reads was recalibrated by associated software, and then duplicated reads were removed using SAMtools 3^18^. Unique mapping reads were then applied for variation detection, and were annotated by RefSeq according to the manufacturer’s recommendation. The variants retrieved from NGS were annotated using an in-house functional prediction tool and then compared with the dbSNP132 and the 1000 Genomes databases. One probable gene variant annotated to *NR0B1* was acquired from our NGS results and was further verified by Sanger sequencing. PCR products were amplified using predesigned primers and purified with a commercial DNA fragment purification kit (Takara, Japan). These amplified PCR fragments were cloned into pUC19 and then analyzed by Sanger sequencing. The hydrophobicity of the resulting wild-type and mutant proteins was predicted using the online tool Protscale (https://web.expasy.org/protscale/) on the ExPASy Server. Additionally, the 3D structure of wild-type and mutant DAX-1 protein was visualized and analyzed using the online tool SWISS-MODEL (https://swissmodel.expasy.org/)^19^.

### Cell culture and transfection

Human adrenal NCI-H295R cells were obtained from the American Type Culture Collection (ATCC) and cultivated in Dulbecco’s modified Eagle’s medium (DMEM; Gibco, USA) supplemented with 10% fetal bovine serum (FBS; Gibco, USA). Cells were cultured at 37 °C in a humidified 5% CO_2_ atmosphere suppled with penicillin and streptomycin (Gibco, USA). Before transfection, cells were seeded in a 24-well plate for 24 h. In total, 60 ng of WT or mutant DAX-1 plasmid was transfected into these seeded cells using Lipofectamine 2000 (Invitrogen, USA) according to the manufacturer’s standard protocols. In addition, siRNA of PPARG1A and mutant DAX-1 plasmid were simultaneously transfected into seeded cells. Thereafter, positive transfected cells were screened and selected on plates containing DMEM supplemented with G418 for further experimental analyses.

### Luciferase reporter assays

A human *NR0B1* gene fragment was first amplified by PCR. Subsequently, the PCR fragments were purified and inserted into the pcDNA3.1 expression vector. Site-directed mutagenesis was performed to generate the DAX-1 mutant according to previous reports^20^. DNA sequencing was used to confirm the correct sequence of both the wild-type and variant.

The regulatory role of *NR0B1* in modulating transcription was monitored using the StAR-luc plasmid (luciferase reporter plasmid) following the method outlined in a previous report^21^. Briefly, seeded cells were treated with 20 ng StAR-luc, 0.5 ng pRL-SV40, and 60 ng WT or mutant *NR0B1*. Next, 20 ng NR5A1 plasmid encoding SF-1 was added to the seeded cells using Lipofectamine 2000 (Invitrogen, USA) according to the manufacturer’s standard protocols. The luciferase activity in the transfected cells was determined using the Dual-Glo luciferase assay system (Promega, USA). Data were normalized to Renilla luciferase activity.

### ChIP-seq assays

The ChIP-seq assay was performed by BGI (Shenzhen, China) to identify the genes associated with mutant DAX-1. In brief, cells treated with mutant DAX-1 were fixed with 1% formaldehyde for 5 min at room temperature and glycine was added to stop the crosslinking reaction. Cells were lysed in ChIP lysis buffer with a sonication system. After dilution with mixing buffer (20 mM Tris–HCl, 150 mM NaCl, 2 mM EDTA, and 1% Triton X-100), the sheared chromatin was subjected to immunoprecipitation with an anti-*NR0B1* antibody (Abcam, USA). The immunoprecipitated DNA was then harvested and subjected to several washes with ChIP-RIPA buffer, high-salt ChIP-RIPA buffer, LiCl buffer, and finally TE buffer. Following treatment, the DNA fragments were purified using a commercial DNA purification kit. For ChIP-seq, ChIP-seq libraries were prepared using the NEBNext DNA sample prep master mix (NEB, USA) according to manufacturer’s protocol. After validating the quality of the library using a Bioanalyzer 2100 (Agilent, USA), the library was sequenced by NGS on an Illumina HiSeq2000 platform (Illumina, USA). Raw data were processed using Bowtie based on a human genome reference. Thereafter, the mapped reads were screened using Model-based Analysis for ChIP-seq (MACS) to identify ChIP-seq peaks and regions of *NR0B1* enrichment.

### qPCR assays

To determine the expression level of mutant DAX-1-associated genes, RNA was isolated from NCI-H295R transgenic or WT cells using the Qiagen RNeasy Mini Kit (Qiagen, USA) and then transcribed into cDNA using the QuantiTect Reverse Transcription Kit (Qiagen, USA). Quantitative real-time PCR (qPCR) was performed using TB Green Fast qPCR Mix (Takara, Japan) and conducted on a real-time PCR detection system (CFX96, Bio-rad, USA) according to previously mentioned protocols^22^. The relative expression levels of associated genes were determined by the 2^−△△Ct^ method after normalization to the endogenous gene GAPDH. All of the primers used are listed in Supplementary Table 2.

### Hormone determination assays

The activities of cellular or extracellular hormones, including progesterone, testosterone, 17B-estradiol, cytochrome P450 steroidogenic enzyme (SE) CYP11A1, follicle stimulating hormone (FSH), and luteinizing hormone (LH), were determined by enzyme-linked immunosorbent assay (ELISA) using purchased commercial kits following the manufacturers’ procedures.

### Statistical analysis

All experiments in this study were performed to obtain at least three biological replicates, and the retrieved data are expressed as the mean ± SD (standard deviation of the mean). Statistical analyses were performed using GraphPad Prism 8.0 (GraphPad, USA). The retrieved data were subjected to a Kruskal–Wallis one-way Analysis of Variance (ANOVA) to evaluate differences. Different lowercase letters on the bars of columns indicate the significance of differences at *P* < 0.05, while the same lowercase letters on the bars of columns indicate no significant differences at *P* > 0.05 (n.s.).

### Ethics statement

This study was performed in accordance with the ethical guidelines of the Declaration of Helsinki and was approved by the Institutional Review Board of the First Affiliated Hospital at Zhengzhou University (Zhengzhou, China). The statement on informed consent (in both Chinese and English) was obtained from the patient and other participants. The statement on consent to publish the information in this study was obtained from the patient and other participants.

## Results

### Clinical evaluation of the patient with CAH

One Chinese family from Henan Province was recruited for this study. The pedigree of this family was plotted based on our clinical investigation, and revealed an X-linked recessive inheritance pattern. This family spans three generations, with five heathy living members and one family member living with CAH (Fig. 1A). We found that the proband, a 24-year-old male, presented with skin hyperpigmentation, fatigue, vertigo, nausea, emaciation, vomiting, and poor sleep according to a clinical examination and the patient’s description. The patient was diagnosed with typical signs of adrenal abnormality and precocious puberty at 20 years of age. The patient and his parents declared that there was no reported history of surgery or additional diseases. With the exception of these symptoms, our clinical evaluation found no other pathologic findings that could account for the presence of endocrine disorders. In addition, the patient’s hormone levels remained disordered after therapy with hydrocortisone and fludrocortisone. Specifically, his adrenotropic hormone levels were high (120 pg/mL), while the levels of testosterone (< 0.13 ng/mL), luteinizing hormone (0.09 U/mL), and renin (0.86 ng/mL) levels were found to be lower than normal, and potassium was in the normal range (4.08 mmol/mL). The patient’s parents were non-consanguineous and reported no history of hypertension or recurrent episodes of periodic paralysis. In addition, none of the other family members suffered from other related endocrine or systemic syndromes.

### Identification of a single novel nonsense variant in the patient with CAH

The exonic sequences of endocrine-related genes and other genes were captured and sequenced using genomic DNA obtained from blood samples. Nearly 539.72 Mb of raw data and 505.48 Mb of processed data were generated from our NGS results. More than 95% mean coverage of target regions was obtained in this NGS dataset, with an average sequencing depth of > 400 × . Furthermore, the coverage of target bases for the N10 and N20 reads from this dataset were 86.8% and 75.9%, respectively. One variant greater than 0.05 existed after filtration by minor allele frequency from an in-house Asia database. This was then validated by Sanger sequencing. In silico analysis revealed one novel nonsense variant in the *NR0B1* gene that has not been previously reported or recorded. In this family, a nonsense variant was identified in the first exon of *NR0B1* (c.265C>T), which resulted in the substitution of a conserved glutamine (Gln) with a stop codon (Ter) at codon 89 (p.Gln89*). We further sought to verify the occurrence of this variant in all affected members by Sanger sequencing (Fig. 1B). The mother and sister of the patient were heterozygotes for this variant, whereas the variant was absent in all unaffected male family members and also healthy controls. Accordingly, this novel variant was not reported in other studies. Additionally, this mutation was evaluated to be causative for CAH formation.

### Bioinformatic assessment demonstrated that the *NR0B1* variant was deleterious

It has been reported that the function and structure of DAX-1 can be influenced by a resultant truncated polypeptide caused by a variant^23^. Therefore, we first evaluated the impaired structure of our variant using SWISS-MODEL. After simulation, we found that this novel variant disrupted the entire amino acid order so that the mutant structure could not be built using SWISS-MODEL. Additionally, nearly 81% of the structure was missing compared to wild-type DAX-1, implying that this variant could potentially disrupt the function of DAX-1. In addition, the hydrophobicity of wild-type and mutant DAX-1 predicted by Protscale demonstrated that this variant largely altered the hydrophobicity of mutant DAX-1 (Supplementary Figure 1). Collectively, the in silico data showed the deleterious impact of this novel variant, which has the potential to cause CAH.

### Transcriptional initiation was suppressed by the *NR0B1* variant

**Figure 2:**
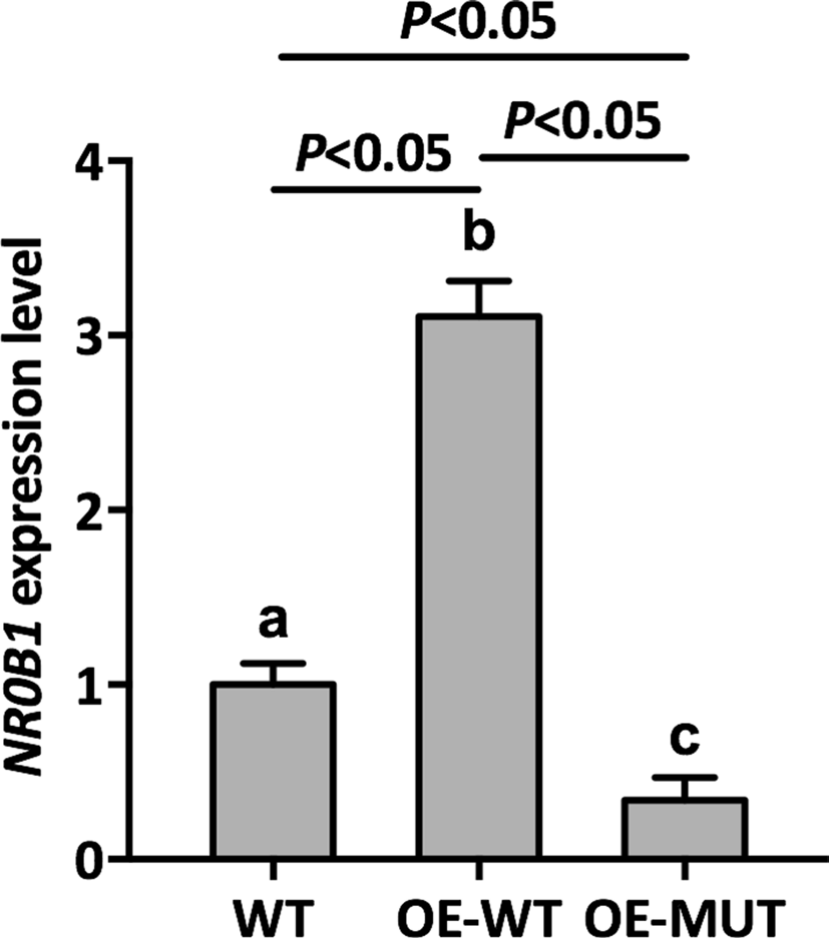
Relative *NR0B1* expression level in transfected cells and non-transfected cells. WT: Wild-type cells, OE-WT: Cells overexpressing normal *NR0B1*, OE-MUT: Cells overexpressing variant *NR0B1*. Error bars represent the standard deviation of triplicate samples. Different lowercase letters represent the significance of differences at *P* < 0.05.

To investigate the influence of this nonsense variant on *NR0B1* transcription, plasmids containing *NR0B1* and its variant were transiently transfected into NCI-H295R cells. Relative *NR0B1* expression levels were then determined by qPCR. As shown in Fig. 2, the *NR0B1* expression levels in mutant DAX-1 cells were remarkably decreased compared to WT, whereas wild-type DAX-1 overexpression resulted in significant elevation of relative *NR0B1* transcripts compared to control and mutant cells, suggesting that the in vivo transcriptional abundance of *NR0B1* was suppressed due to this variant. This finding also demonstrated that mutant *NR0B1* could suppress normal *NR0B1* transcriptional levels.

**Figure 3:**
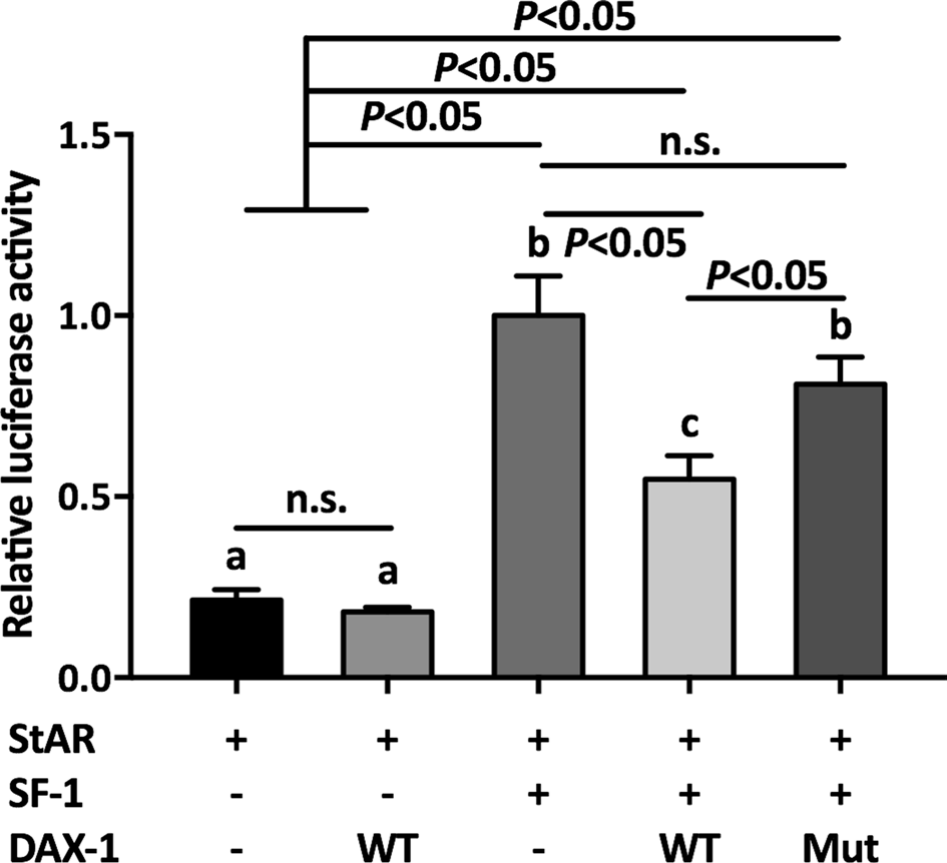
Luciferase assay for SF-1 mediated StAR transactivation in the presence of wild-type or mutant DAX-1. StAR: Steroidogenic acute regulatory protein, SF-1: Steroidogenic factor 1, WT: Wild-type DAX-1 plasmid, Mut: Mutant DAX-1 plasmid. Error bars represent the standard deviation of triplicate samples. Different lowercase letters represent the significance of differences at *P* < 0.05.

Considering the crucial role of *NR0B1* as a transcriptional factor that regulates the transcriptional activation of its target genes, we next sought to verify the transcriptional regulatory role of mutant DAX-1. DAX-1 has been proven to function as a negative regulator to suppress SF-1-mediated transactivation of steroidogenic genes, such as StAR^24^. Hence, in this study, we performed luciferase assays to investigate the impairment of original DAX-1 function (Fig. 3). As expected, wild-type DAX-1 suppressed the relative luciferase activity, which was in accordance with the finding of a previous report^21^. Intriguingly, mutant DAX-1 significantly improved luciferase activity compared to wild-type DAX-1, suggesting that this *NR0B1* variant impaired the repressor function of DAX-1 during transcriptional regulation. Moreover, compared to the group without wild-type DAX-1, the luciferase activity of mutant DAX-1 was relatively lower, demonstrating that DAX-1 still had partial function with this novel variant.

### Variant *NR0B1* regulated several genes associated with body-metabolism

**Figure 4:**
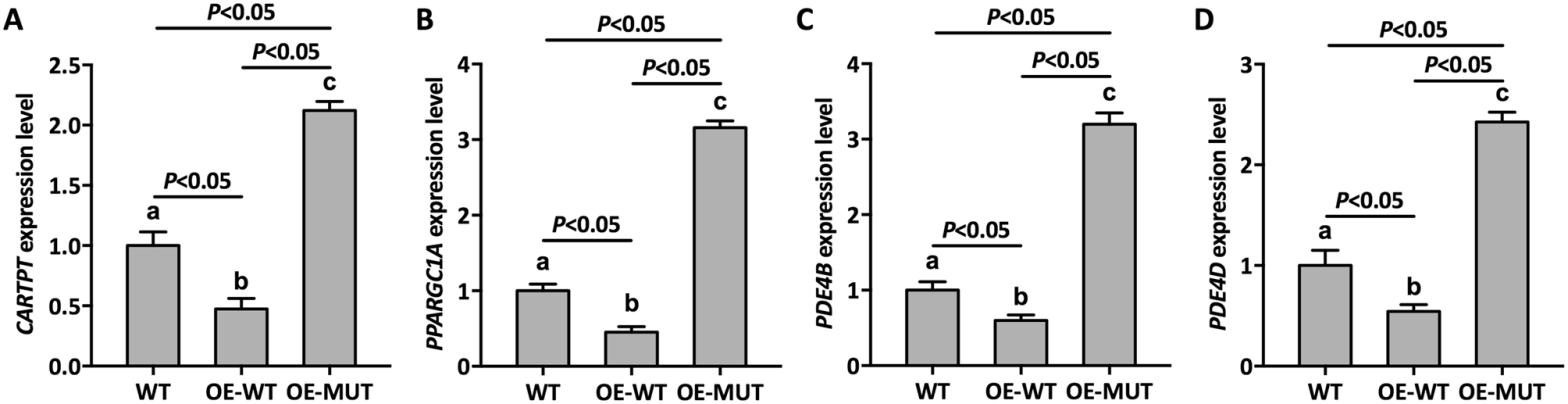
Relative expression level of *NR0B1*-associated genes in transfected and non-transfected cells. (**A**) *CARTPT*, (**B**) *PPARGC1A*, (**C**) *PDE4B*, and (**D**) *PDE4D*. WT: Wild-type cells, OE-WT: Cells overexpressing normal *NR0B1*, OE-MUT: Cells overexpressing variant *NR0B1*. Error bars represent the standard deviation from triplicate samples. Different lowercase letters represent the significance of differences at *P* < 0.05.

Although this nonsense variant of *NR0B1* impaired self-transcriptional regulation based on the above data, the molecular mechanism underlying CAH remains obscured in this particular case. To this end, in order to further elucidate the influence of this *NR0B1* variant on targeting genes, we preformed ChIP-sequence analysis of *NR0B1* variants with an anti-*NR0B1* polyclonal antibody in NCI-H295R human adrenocortical tumor cells. After sequencing, we obtained numerous reads distributed in several chromosomes (Supplementary Figure 2). Moreover, numerous pathways were found to be perturbed in mutant DAX-1 compared to WT (Supplementary Figure 3 and 4). Four typical genes, including *CARTPT*, *PPARGC1A*, *PDE4B*, and *PDE4D*, were found to be closely related to *NR0B1* as shown in Supplementary Table 1. Interestingly, the loci of these four genes were not near the loci of *NR0B1*, and, in some cases, were even from a different chromosome. Among these genes, *CARTPT* seems to have anorexigenic effects based on previous literature^25^. To validate the regulation of *CARTPT* by DAX-1, qPCR was performed using the primers shown in Supplementary Table 2. As shown in Fig. 4A, the DAX-1 mutant caused *CARTPT* upregulation compared to wild-type DAX-1, which confirmed that this *NR0B1* variant could mediate *CARTPT* upregulation. This may have caused anorexigenic effects for the patient, leading to emaciation, which was congruent with the patient’s clinical record. In addition, the relative transcript abundance of *PPARGC1A* gene (encoding peroxisome proliferator-activated receptor gamma coactivator 1 alpha), a potentially powerful coactivator of many transcriptional factors related to steroidogenesis, was then evaluated. As shown in Fig. 4B, the expression of *PPARGC1A* was upregulated by mutant DAX-1, which may have resulted in abnormal steroidogenesis and energy homeostasis, finally leading to CAH. Phosphodiesterases (PDEs) can regulate the intracellular levels of cAMP, the most important regulatory signaling molecule in the control of steroidogenesis, and can affect steroidogenesis. Our results confirmed that the expression of *PDE4B* and *PDE4D* was upregulated by mutant DAX-1 (Fig. 4C,D), which in turn, impaired steroidogenesis; this was consistent with the patient’s clinical data.

### DAX-1 mutant stimulated hormone disorders

**Figure 5:**
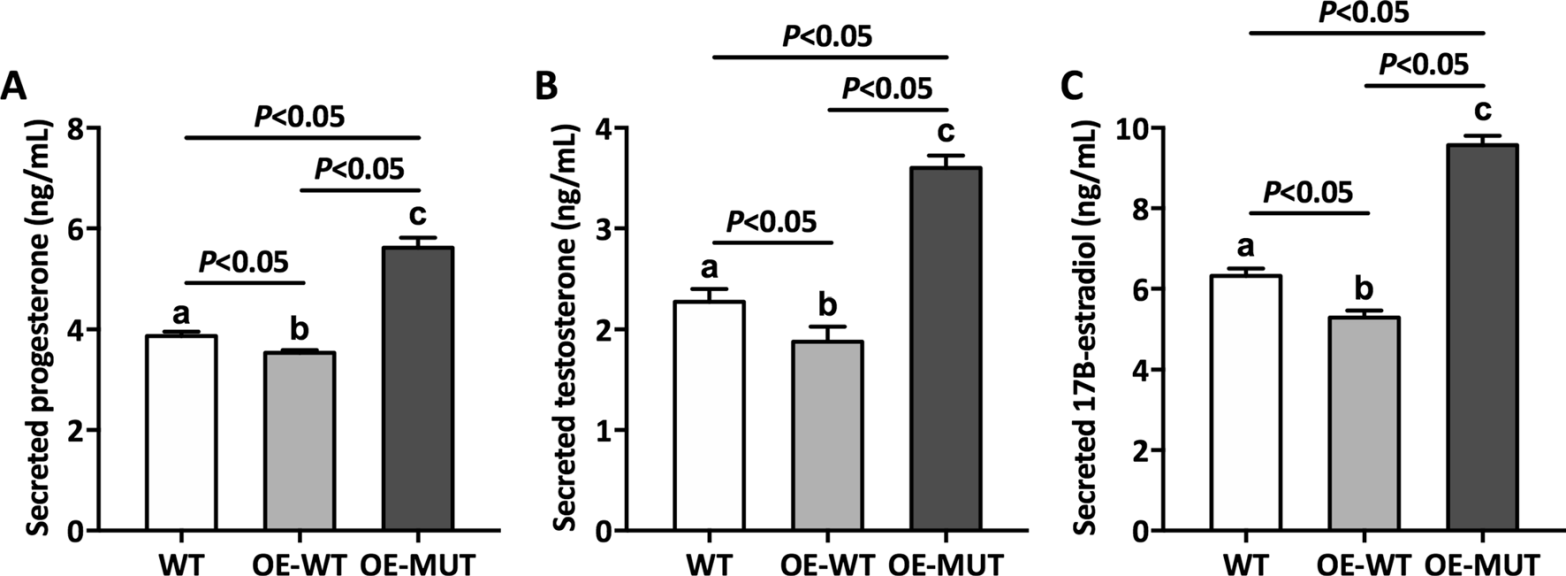
Secreted hormone levels of transfected and non-transfected cells. (**A**) Secreted progesterone level, (**B**) Secreted testosterone level, and (**C**) Secreted 17B-estradiol level. WT: Wild-type cells, OE-WT: Cells overexpressing normal *NR0B1*, OE-MUT: Cells overexpressing variant *NR0B1*. Error bars represent the standard deviation from triplicate samples. Different lowercase letters represent the significance of differences at *P* < 0.05.

**Figure 6:**
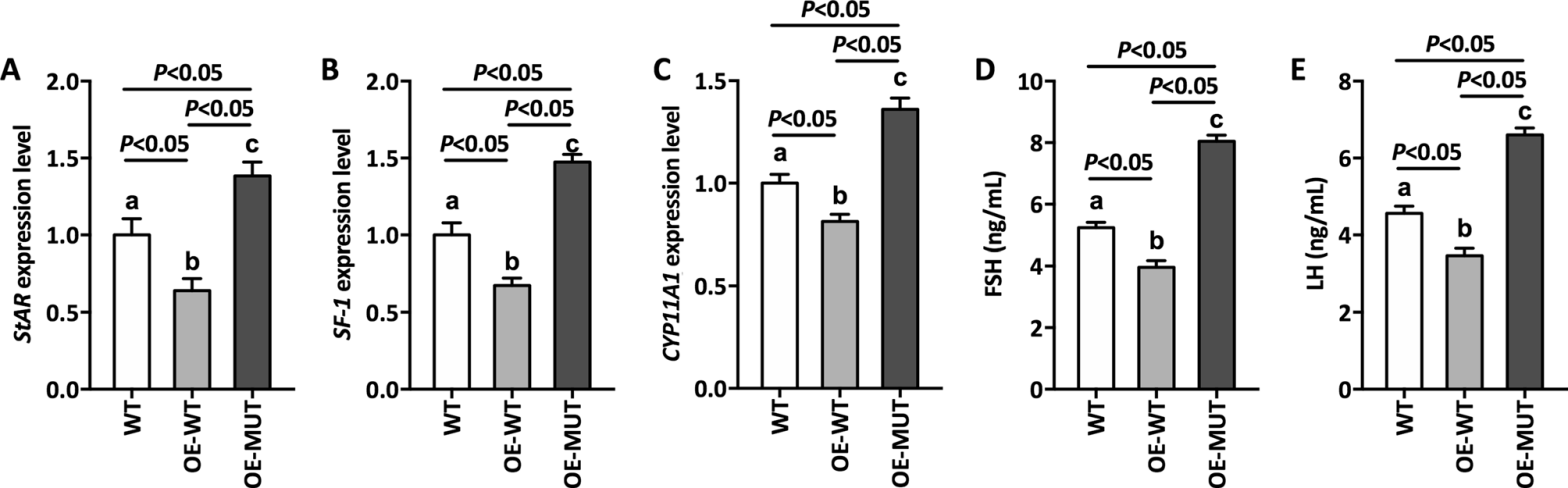
The levels of typical transcriptional factor and hormone of transfected and non-transfected cells. (**A**) StAR, (**B**) SF-1, (**C**) CYP11A1, (**D**) FSH, and (**E**) LH. StAR: Steroidogenic acute regulatory protein, SF-1: Steroidogenic factor 1, CYP11A1: Cytochrome P450 steroidogenic enzyme CYP11A1, FSH: Follicle stimulating hormone, LH: Luteinizing hormone. Error bars represent the standard deviation from triplicate samples. Different lowercase letters represent the significance of differences at *P* < 0.05.

The hormone levels of our patient were abnormally expressed, which we speculated was caused by the nonsense variant. To test this, the changes in hormone levels caused by the DAX-1 mutant were determined for several common hormones. As shown in Fig. 5, overexpression of wild-type *NR0B1* (OE-WT) caused the downregulation of secreted hormones, including progesterone, testosterone, and 17B-estradiol, suggesting that wild-type *NB0B1* expression caused this hormone change. Predictably, after overexpression of variant *NR0B1* (OE-MUT), the levels of secreted hormones increased compared to those of the WT and OE-WT groups. In addition, the activities of StAR and SF-1 were enhanced by variant *NR0B1* overexpression compared to the other two groups (Fig. 6A,B), demonstrating that mutant DAX-1 could directly regulate StAR and SF-1 in hormone disorders, which was in accordance with our hormone data in Fig. 5. Furthermore, the level of the cytochrome P450 steroidogenic enzyme CYP11A1 was significantly upregulated in OE-MUT (Fig. 6C), implying that our DAX-1 mutant regulated related target genes (such as *PPARGC1A* and PDEs), resulting in a disorder of steroidogenesis. It is well known that the unordered expression levels of FSH and LH are frequently detected in patients with CAH. Therefore, it became necessary to detect the corresponding hormones in cells overexpressing variant *NR0B1*. As expected, FSH and LH reached maximum values of 8.05 and 6.61 ng/mL in the OE-MUT line, respectively (Fig. 6D,E), which suggested that the high expression levels of FSH and LH were caused by this *NR0B1* variant.

### *PPARGC1A* inhibition caused the recovery of hormone levels in the DAX-1 mutant

**Figure 7:**
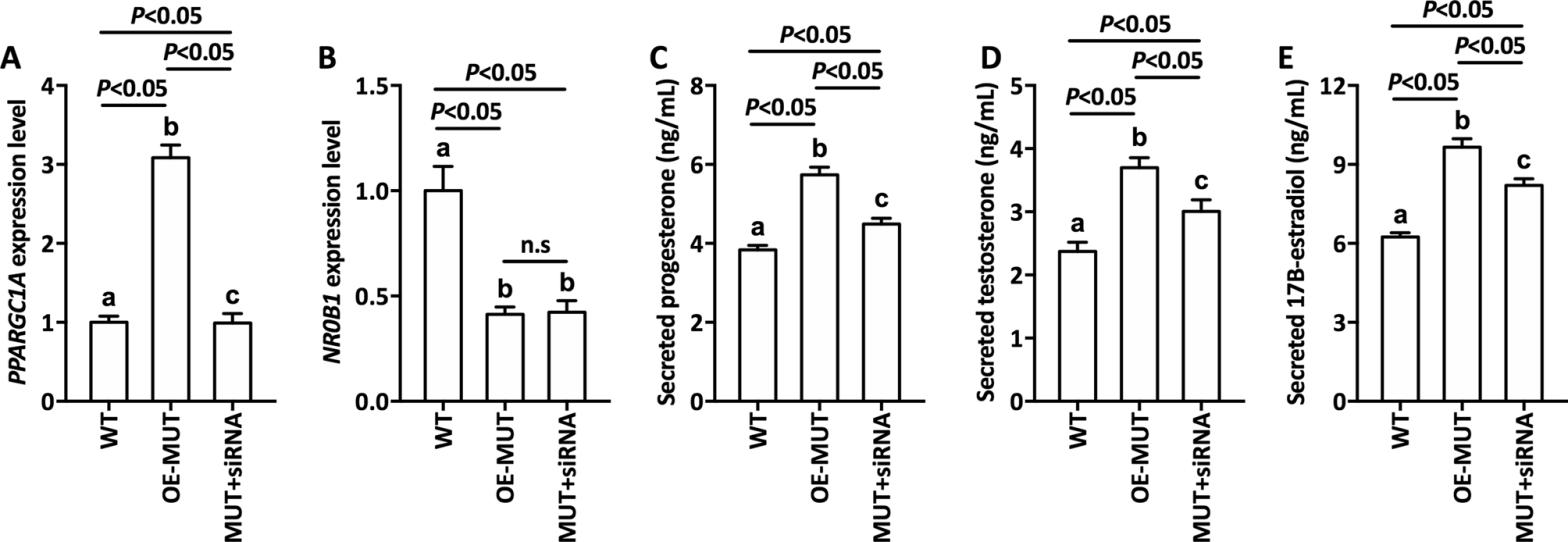
Biochemical characteristics of cells treated with siRNA of PPARGC1A. (**A**) Relative expression level of *PPARGC1A* and (**B**) *NR0B1*. (**C**) Secreted progesterone level, (**D**) secreted testosterone level, and (**E**) secreted 17B-estradiol. WT: Wild-type cells, OE-WT: Cells overexpressing normal *NR0B1*, MUT-siRNA: Cells overexpressing variant *NR0B1* and siRNA of PPARGC1A. Error bars represent the standard deviation from triplicate samples. Different lowercase letters represent the significance of differences at *P* < 0.05.

The expression of *PPARGC1A*, which was identified as being pivotal in steroidogenesis, was upregulated by variant *NR0B1* based on our qPCR data. We also explored the related physiological parameters of variant *NR0B1* by adding siRNA targeting *PPARGC1A*. As expected, *PPARGC1A* expression levels were downregulated to the level of WT, suggesting that siRNA of *PPARGC1A* was successfully integrated into these cells to influence the expression of *PPARGC1A* (Fig. 7A). Intriguingly, the *NR0B1* expression level was unchanged after the expression of siRNA of PPARGC1A (Fig. 7B). In addition, the expression of secreted hormones (Fig. 7C-E) was partially inhibited to a relatively low level by the siRNA of PPARGC1A, confirming that variant *NR0B1* regulates *PPARGC1A* to modulate hormone expression and secretion.

## Discussion

In this study, for the first time, we identified a novel nonsense variant located in *NR0B1* with X-linked inheritance from a three-generation Han family by NGS analysis, which was further corroborated by Sanger sequencing (Fig. 1). Our data showed that the particular novel nonsense variant was co-segregated in a proband in this pedigree, while no such variant was observed in healthy male family members and other healthy individuals. These results demonstrate that this nonsense variant may be responsible for the CAH phenotype with X-linked inheritance in a Chinese Han family. It is well known that X-linked CAH is caused by a DAX-1 variant, which is encoded by *NR0B1* composed of two exons separated by one intron. Numerous additional variants have been identified since the discovery of *NR0B1* as the gene responsible for CAH. Currently, approximately 200 variants of *NR0B1* have been reported in patients with CAH, all of which alter the C-terminus of the DAX-1 protein. Among them, nearly 50% are frameshift variants, which cause the loss of the repressive functions of DAX-1. It has been reported that the truncated protein caused by in-dels might affect the transcriptional repression and post-transcriptional regulation of this gene, resulting in the occurrence of CAH^26^. Interestingly, in this case, we identified a novel nonsense *NR0B1* variant in a Han population; this variant has a truncated protein lacking the C-terminal ligand binding domain, implying that the deleterious effects were due to the lack of this C-terminal ligand binding domain. This gene variant was predicted to cause crucial structural changes in the DAX-1 protein, with nearly 81% of the protein structure missing compared to wild-type DAX-1, thereby disrupting the function of mutant DAX-1. The loss of DAX-1 function triggered by this *NR0B1* variant caused disordered expression of genes involved in energy homeostasis and steroidogenesis, which may lead to abnormal adrenal development and finally CAH. These findings are in line with previous reports in this field^21,23,26^.

To verify this notion with regards to the deleterious impacts of mutant DAX-1, functional analyses were performed to evaluate the consequences of this novel nonsense variant. The qPCR results shown in Fig. 2 demonstrate that overexpression of mutant DAX-1 significantly downregulated the expression of *NR0B1* compared to WT, suggesting that mutant DAX-1 represses *NR0B1* transcription in vivo.

It has been reported that DAX-1 plays a crucial role in steroid and hormone biosynthesis as a transcriptional factor^27–29^. Related literature has shown that DAX-1 is a negative regulator that represses the SF-1 (steroidogenic factor 1)-mediated transactivation of various key genes related to steroidogenesis^24^. Two such variants (Y380D and I439S) in DAX-1 were found to be located in the C-terminal region of the protein, and showed a partial repression activity of SF-1-mediated transcription^30,31^. Rojek et al*.* demonstrated a novel hemizygous deletion encompassing the entire *NR0B1* gene together with the *MAGEB* genes, which was associated with congenital adrenal insufficiency and normal development of male external genitalia^32^. Intriguingly, a Q37X nonsense variant in DAX-1 has been reported to cause a truncated isoform of DAX-1 protein which exhibited partial residual activity, thereby delaying the onset of adrenal insufficiency until early adulthood^33^. The current patient suffered from CAH from early adulthood, suggesting that this mutant DAX-1 possessed partial residual activity, which may have delayed the disorders of hormone biosynthesis in this patient. We next performed a luciferase assay to elucidate the effects of a mutant on the repressor function of DAX-1. As expected, mutant DAX-1 impaired the function of DAX-1 with a higher luciferase activity compared to WT, which demonstrated the abnormal regulation of mutant DAX-1 (Fig. 3). Numerous previous studies have focused on the traditional regulatory mechanism involving the DAX-1 mutant. To elucidate the molecular mechanism of this novel nonsense variant, we first performed ChIP-seq to identify target genes associated with mutant DAX-1. Interestingly, four crucial genes, including *CARTPT*, *PPARGC1A*, and *PDE4*, were found to be associated with mutant DAX-1. In addition, related secreted hormones and regulation factors were detected, suggesting that mutant DAX-1 caused disordered hormone and regulation factor expression (Figs. 5, 6). CARTPT encodes a preproprotein that is proteolytically processed to generate multiple biologically active peptides involved in appetite, energy balance, body weight maintenance, and stress response. Lack of *CARTPT* in mice has been shown to result in increased body weight, and viral delivery of *CARTPT* mRNA through the intracerebroventricular cannula suppressed weight gain in diet-induced obese rats^34–36^. Interestingly, in our case, CARTPT was upregulated by the *NR0B1* variant compared to WT, demonstrating that this mutant DAX-1 controlled the body weight via disordered *CARTPT* expression, which was in accordance with the findings of a pervious study^37^. Moreover, knockdown of *PPARGC1A* inhibited the gene expression pattern related to testosterone synthesis, leading to disorders of testosterone secretion^38^. Our ChIP-seq data revealed that *PPARGC1A* is a potential target gene related to *NR0B1* that may finally lead to CAH. These findings suggest that *NR0B1* variation triggered the disorder of coactivator *PPARGC1A* and led to unusual energy and testosterone metabolism, which was in accordance with our qPCR data (Fig. 4). Furthermore, we used siRNA of *PPARGC1A* to validate that knockdown of PPARGC1A mutant *NR0B1* partially recovered hormone biosynthesis, implying that interference of *PPARGC1A* regulation could effectively recover cellular hormone stasis (Fig. 7). Additionally, two typical PDE genes, *PDE4B* and *PDE4D*, were found to be related to *NR0B1*, suggesting that abnormal expression of PDE genes influenced steroidogenesis and may cause CAH. It has been shown that inhibition of the enzymes PDE4 and PDE8 significantly increased testosterone levels in Leydig cells^39^, which is in agreement with our data. Previous reports have already demonstrated that chronic ablation of PDE8B increases cAMP-dependent transcription of several key steroidogenic proteins, such as StAR protein and p450scc^40^. It is worth noting that the PDEs in this study were altered by the DAX-1 mutant to affect steroidogenesis, possibly by regulating StAR activity. Therefore, this is a worthwhile hypothesis for us to investigate in future research.

In conclusion, the present study identified a novel nonsense variant of *NR0B1* that crucially regulated several targeted genes, causing inhibition of steroidogenesis, and finally leading to abnormal adrenal development. Our data provide novel insights into the potential mechanism of CAH formation caused by this nonsense variant. Additionally, our findings significantly advance the current understanding of the Chinese variation spectrum of CAH.

## Supplementary Information

Supplementary Information.
