# Supplementary material for: Nonsense variant of NR0B1 causes hormone disorders associated with congenital adrenal hyperplasia
Source: Sci Rep. 2021 Aug 9;11:16066. doi: 10.1038/s41598-021-95642-y (PMC8352982; doi:10.1038/s41598-021-95642-y)
Supplement: Supplementary file 1 — Supplementary Information. [file 41598_2021_95642_MOESM1_ESM.docx]

**Nonsense variant of *NR0B1* causes hormone disorders associated with** **congenital adrenal hyperplasia**

Da-Bei Fan^+1,^*, Li Li^+2^, Hao-Hao Zhang^1^

^1^Endocrine Department, the First Affiliated Hospital of Zhengzhou University, Zhengzhou 450052, China;

^2^Ophthalmologic Center, the First Affiliated Hospital of Zhengzhou University Zhengzhou 450052, China

*Correspondence to D. Fan, email: fccfandb@zzu.edu.cn

^+^ These authors contributed equally to this work.


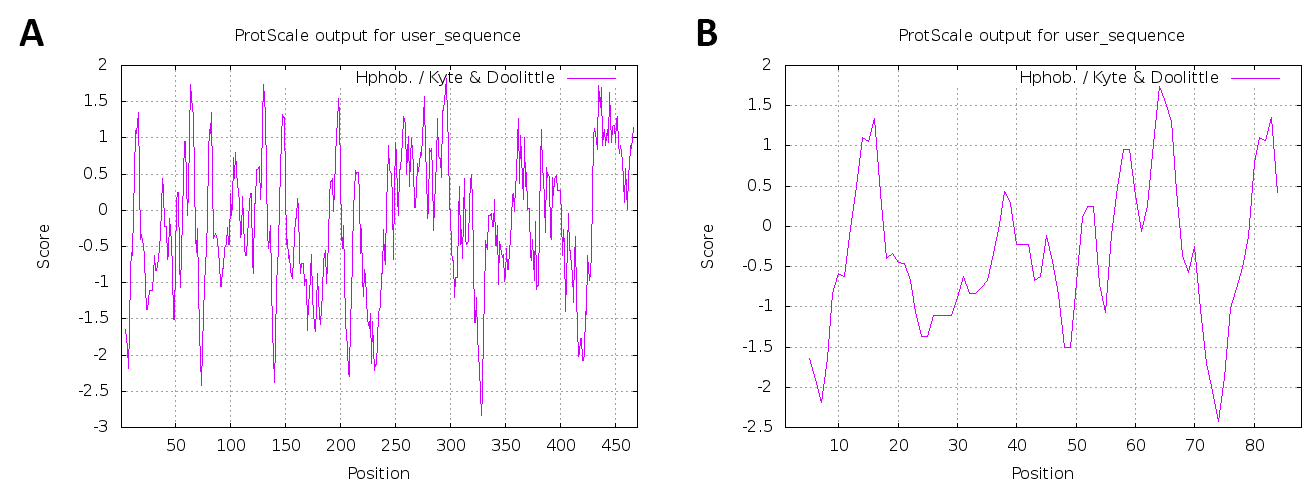


**Supplementary Figure 1** Hydrophobicity of wild-type and mutant DAX-1. (A) Wild-type DAX-1, (B) Mutant DAX-1 caused by nonsense variant (c.265C>T).


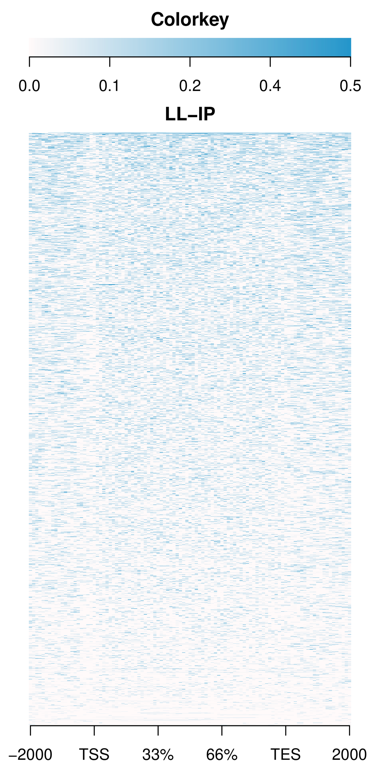


**Supplementary Figure 2** Heat map of read distribution retrieved from ChIP-seq by nonsense mutation of DAX-1.


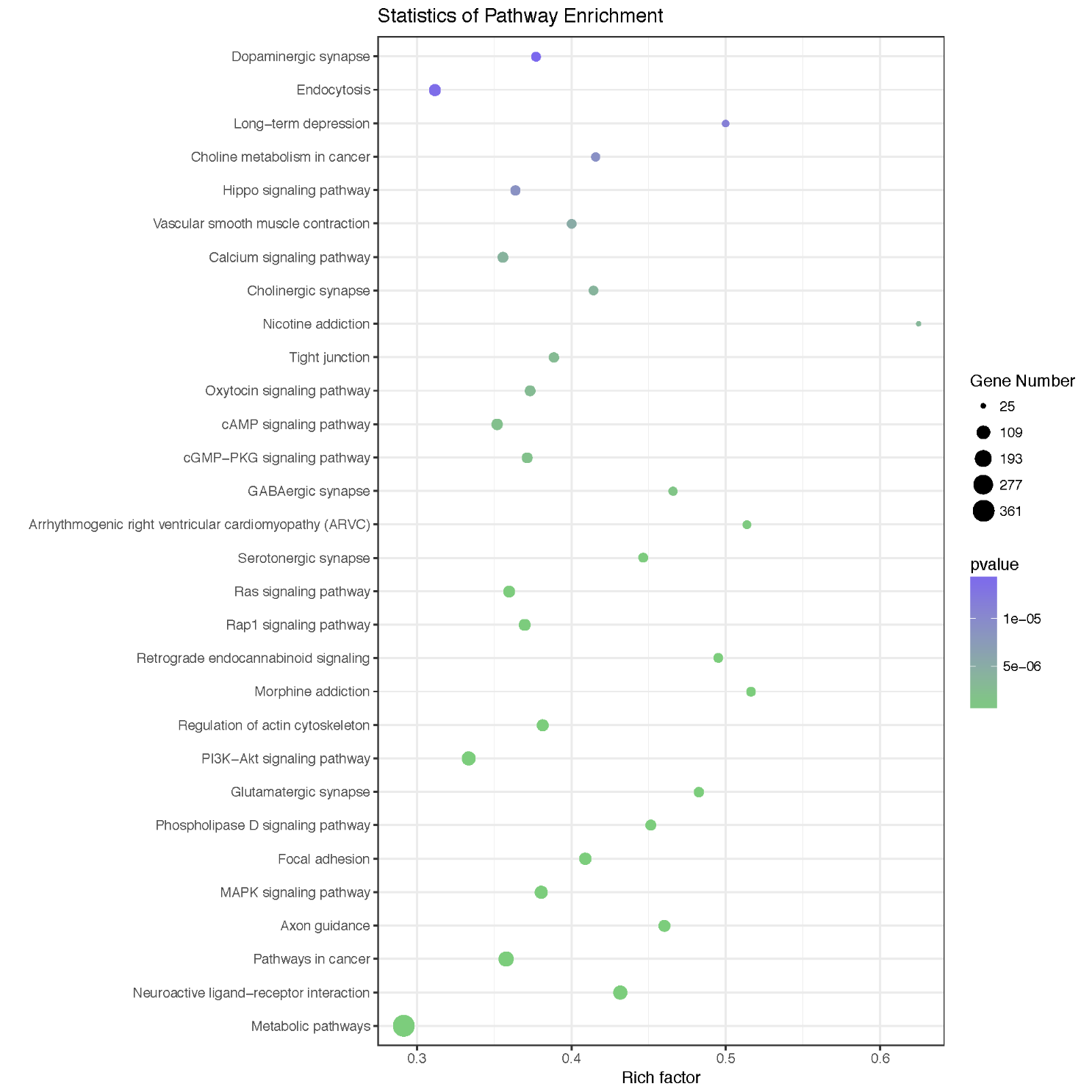


**Supplementary Figure 3** Classification chart of associated genes in KEGG pathways retrieved from ChIP-seq by nonsense mutation of DAX-1.


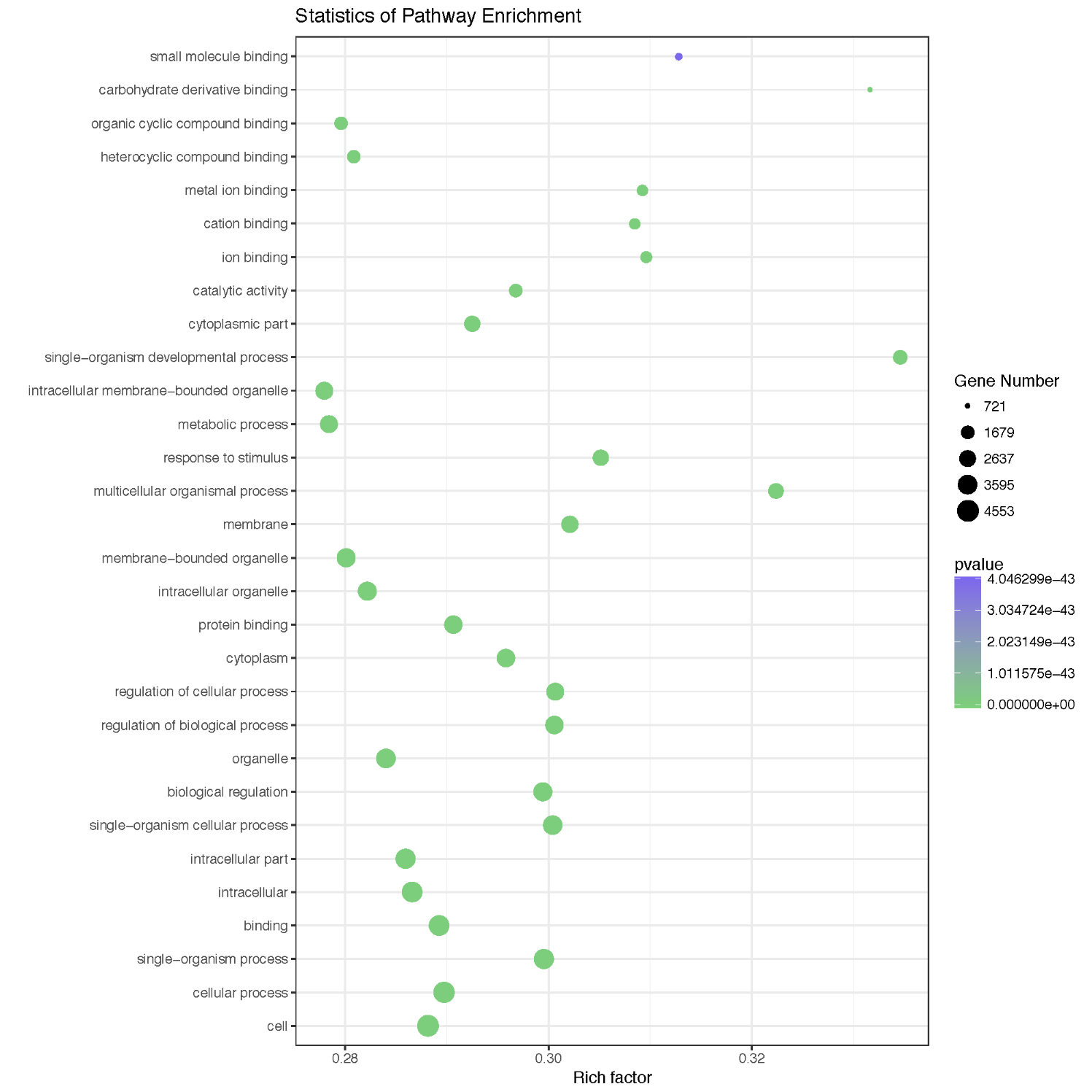


**Supplementary Figure 4** Enrichment analysis of associated genes according to gene ontology analysis retrieved from ChIP-seq by nonsense mutation of DAX-1.


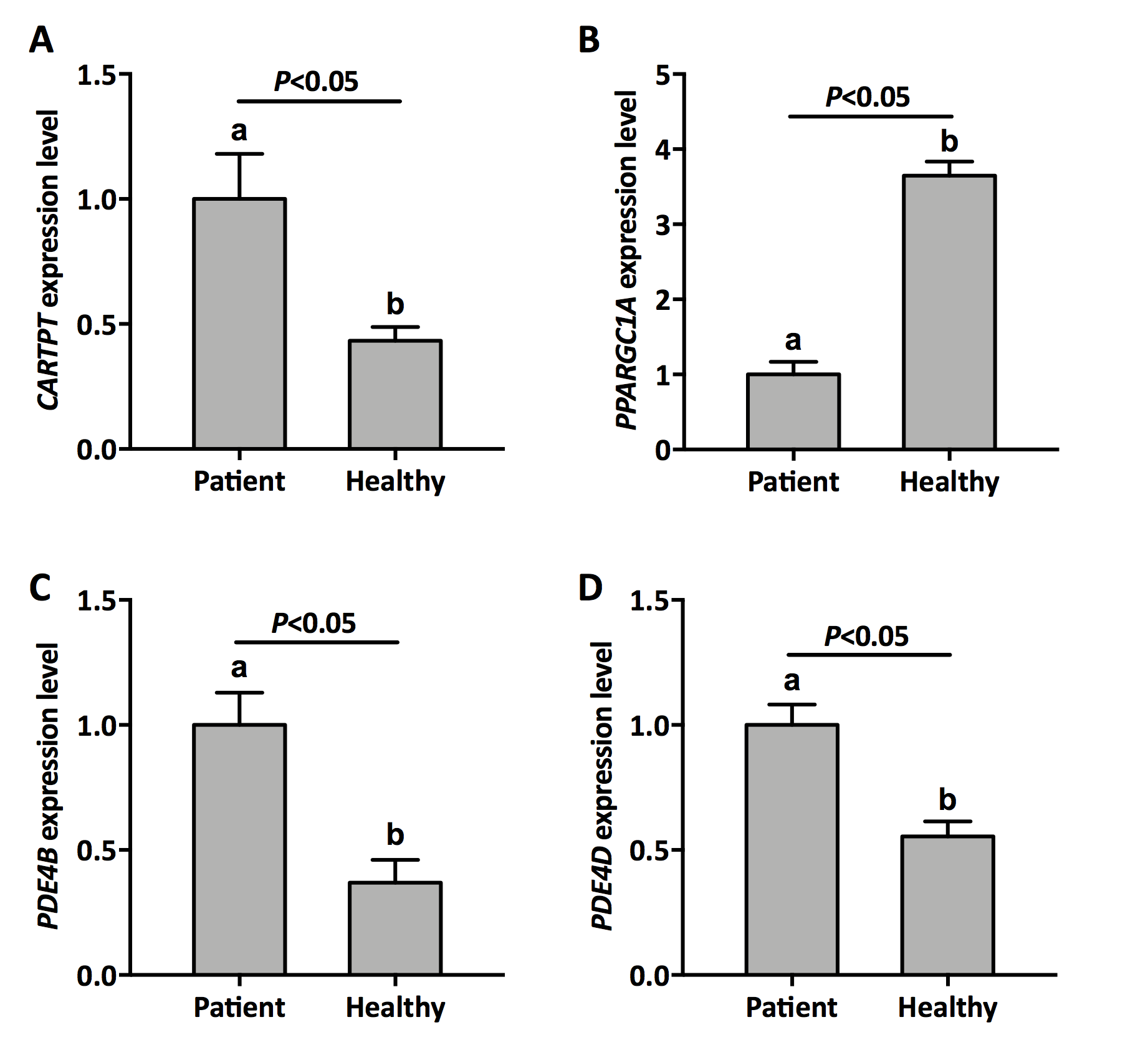


**Supplementary Figure 5** Relative expression level of *NR0B1*-associated genes in patient (proband) and healthy blood samples. (A) *CARTPT*, (B) *PPARGC1A*, (C) *PDE4B*, and (D) *PDE4D*. Error bars represent the standard deviation of triplicate samples. Different lowercase letters represent the significant difference at *P* < 0.05.

**Supplementary Table 1** Classification chart of associated genes in KEGG pathways retrieved from ChIP-seq by nonsense mutation of DAX-1.

| Chr | Region | Transcript | Gene | Gene ID | Description | Type |
| --- | --- | --- | --- | --- | --- | --- |
| chr5 | intergenic | NM_004291.3 | CARTPT | 9607 | CART prepropeptide | protein-coding |
| chr4 | intergenic | XM_005248133.1 | PPARGC1A | 10891 | peroxisome proliferator-activated receptor gamma-2C coactivator 1 alpha | protein-coding |
| chr1 | intergenic | NM_001037341.1 | PDE4B | 5142 | phosphodiesterase 4B | protein-coding |
| chr5 | intron | XM_005248539.1 | PDE4D | 5144 | phosphodiesterase 4D | protein-coding |

**Supplementary Table 2** The primers of qPCR performed in this study.

| Gene | Transcript | Primer name | Sequence (5’⭢3’) |
| --- | --- | --- | --- |
| CARTPT | NM_004291.3 | cartpt-f | ATCTACTCTGCCGTGGAT |
|  |  | cartpt-r | TGAGCTTCTTCAAGACTTCT |
| PPARGC1A | XM_005248133.1 | ppargc1a-f | GATGACAGCGAAGATGAA |
|  |  | ppargc1a-r | GAAGAACAAGAAGGAGACA |
| PDE4B | NM_001037341.1 | pde4b-f | AACAATACAAGCATCTCA |
|  |  | pde4b-r | GAATATCCAGCCACATTA |
| PDE4D | XM_005248539.1 | pde4d-f | CAGAATAGCAGAGTTGTC |
|  |  | pde4d-r | ATGGATATTGTTGTGATAGG |
